# Exploring the variables influencing the immune response of traditional and innovative glycoconjugate vaccines

**DOI:** 10.3389/fmolb.2023.1201693

**Published:** 2023-05-16

**Authors:** Francesca Micoli, Giuseppe Stefanetti, Calman A. MacLennan

**Affiliations:** ^1^ GSK Vaccines Institute for Global Health, Siena, Italy; ^2^ Department of Biomolecular Sciences, University of Urbino Carlo Bo, Urbino, Italy; ^3^ Enteric and Diarrheal Diseases, Global Health, Bill and Melinda Gates Foundation, Seattle, WA, United States; ^4^ The Jenner Institute, Nuffield Department of Medicine, University of Oxford, Oxford, United Kingdom; ^5^ The Institute of Immunology and Immunotherapy, University of Birmingham, Birmingham, United Kingdom

**Keywords:** glycoconjugate, carbohydrate, vaccines, immune response, conjugation variables

## Abstract

Vaccines are cost-effective tools for reducing morbidity and mortality caused by infectious diseases. The rapid evolution of pneumococcal conjugate vaccines, the introduction of tetravalent meningococcal conjugate vaccines, mass vaccination campaigns in Africa with a meningococcal A conjugate vaccine, and the recent licensure and introduction of glycoconjugates against *S.* Typhi underlie the continued importance of research on glycoconjugate vaccines. More innovative ways to produce carbohydrate-based vaccines have been developed over the years, including bioconjugation, Outer Membrane Vesicles (OMV) and the Multiple antigen-presenting system (MAPS). Several variables in the design of these vaccines can affect the induced immune responses. We review immunogenicity studies comparing conjugate vaccines that differ in design variables, such as saccharide chain length and conjugation chemistry, as well as carrier protein and saccharide to protein ratio. We evaluate how a better understanding of the effects of these different parameters is key to designing improved glycoconjugate vaccines.

## 1 Introduction

Glycoconjugate vaccines began when Avery and Goebel found that the immunogenicity of polysaccharides (PS) and oligosaccharides (OS) could be enhanced by coupling to carrier proteins and that antibody concentrations increased after reinjection of such conjugates (Avery and Goebel, 1929; Goebel and Avery, 1931; Goebel, 1939). The introduction of glycoconjugate vaccines has greatly reduced the incidence of disease caused by *Haemophilus influenzae* type b (Hib), *Neisseria meningitidis* and *Streptococcus pneumoniae* (Ada and Isaacs, 2003; Trotter et al., 2008). The research on glycoconjugates has never stopped as shown by the evolution of pneumococcal conjugate vaccines (Micoli et al., 2023), the introduction of tetravalent meningococcal conjugate vaccines (Cooper et al., 2011), vaccination campaigns in Africa with the MenAfriVac meningococcal serogroup A vaccine (Frasch et al., 2012) and most recently the licensure of Vi glycoconjugate vaccines against *S.* Typhi (Hancuh et al., 2023).

Many bacteria are surrounded by capsular PS or O-specific polysaccharide (O-SP) which are virulence factors and targets of protective antibodies. The structure of the repeating unit of some of these PS and O-SP are shown in Table 1. Most PS are T-independent type 2 antigens that can directly stimulate B cells without the help of T cells (Mond et al., 1995; Snapper and Mond, 1996; Lesinski and Westerink, 2001). Cross-linking of multiple surface immunoglobulins on B cells by PS activates the B cells which differentiate into antibody-secreting plasma cells. However, PS can be poorly immunogenic, particularly in children below 2 years (Käyhty et al., 1984; Peltola et al., 1984; Austrian, 1989; Peter, 1998). Re-vaccination is often required, since antibody levels decline rapidly, but does not elicit a booster effect indicating that differentiation into memory B cells does not occur (Kelly et al., 2006). Indeed, immunization with unconjugated PS can deplete a preexisting memory B cell pool leading to hyporesponsiveness to subsequent immunization (Granoff and Pollard, 2007; Pollard et al., 2009; Poolman and Borrow, 2011). The antibody response to PS is little affected by adjuvants (Barrett, 1985; Rappuoli, 2018) and only glycoconjugate vaccines have been shown to induce mucosal IgG, which is key to achieve herd immunity.

**TABLE 1:** Polysaccharides for which the impact of certain variables on the immunogenicity of corresponding glycoconjugates has been studied. Some of these saccharides are in licensed vaccines.

Saccharide	Structure	Conjugation variables investigated in preclinical/clinical study	Use in licensed vaccines*
*Clostridium difficile* PS-II	→6)-β-D-Glc*p*-(1→3)-β-D-Gal*p*NAc-(1→4)-α-D-Glc*p*-(1→4)-[β-D-Glc*p*-(1→3]-β-D-Gal*p*NAc-(1→3)-α-D-Man*p*-(1→P	Carrier protein Romano et al. (2014)	
Dextran PS	→6)-α-Glc*p*-(1→	Conjugation chemistry Seppälä and Mäkelä (1989)	
*Escherichia hermannii O-SP*	→3)-β-D-Rha*p*-(1→	Conjugation chemistry Jacques and Dubray (1991)	
*Escherichia coli* O111 O-SP	→4)-[α-D-Col*p*-(1→3 or 6)]-*α*-D-Glc*p*-(1→4) -*β*-D-Gal*p*-(1→3)-*β*-D-Glc*p*NAc-(1→	Conjugation chemistry Gupta et al. (1995)	
*Escherichia coli* K100 O-SP	→3)-*β*-D-Rib*f*-(1→2)-D-Ribitol-(5→OPO_3_→	Carrier protein Chu et al. (1983)	
Group B *Streptococcus* Type II CPS	→2)-[α-D- Neu*p*5Ac-(2→3)]-*β*-D-Gal*p*-(1→4)-*β*-D-Glc*p*NAc-(1→3)-[*β*-D-Gal*p*-(1→6)]-*β*-D-Gal*p*-(1→4)-*β*-D-Glc*p*-(1→3)-*β*-D-Glc*p*-(1→	Carrier protein Nilo et al. (2015a);Nilo et al. (2015b)	
Group B *Streptococcus* Type III CPS	→4)-*β*-D-Glc*p*-(1→6)-[*α*-Neu*p*5Ac (2→3)-β-D-Gal*p*-(1→4)]-*β*-D-Glc*p*NAc-(1→3)-*β*-D-Gal*p*-(1→	Conjugation chemistry Shen et al. (2001); Wessels et al. (1998). Carrier protein Shen et al. (2000);	
Group B *Streptococcus* Type V CPS	→4)-[α-D- Neu*p*5Ac-(2→3)-β-D-Gal*p*-(1→4)-*β*-D-Glc*p*NAc-(1→6)]-*β*-D-Glc*p*-(1→4)-[*β*-D-Glc*p-*(1→3)]-*β*-D-Gal*p-*(1→4)-*β*-D-Glc*p*-(1→	Conjugation chemistry Nilo et al. (2014); Carrier protein Nilo et al. (2015a);Nilo et al. (2015b)	
*Haemophilus influenzae* type b CPS	→3)-*β*-D-Rib*f*-(1→1)-D-Ribitol-(5→OPO_3_→	Saccharide length and saccharide to protein ratio Anderson et al. (1989). Carrier protein Anderson et al. (1985); Falugi et al. (2001); Chu et al. (1983); Schneerson et al. (1984)	ActHIB (Sanofi Pasteur), Hiberix (GlaxoSmithKline Biologicals), Liquid PedvaxHIB (Merck Sharp & Dohme), Pentacel (Sanofi Pasteur), VAXELIS (MSP Vaccine Company)
*Klebsiella pneumoniae* serotype 11 CPS	→3)-[4,6-O-(1-carboxyethylidene)-α-D-Galp-(1→4)]-*β*-D-Glc*p*A-(1→3)-α-D-Gal*p*-(1→3)-*β*-D-Glc*p*-(l→	Carrier protein Zigterman et al. (1985)	
Laminarin PS (used for a conjugate vaccines against *Candida albicans*)	→6)-*β*-D-Glc*p*-(1→3)-*β*-D-Glc*p*-(1→	Carrier protein Romano et al. (2014)	
*Neisseria meningitidis* serogroup A CPS	→6)-α-D-Man*p*NAc(3/4OAc)-(1→OPO_3_→	Saccharide to protein ratio Micoli et al. (2020). Conjugation chemistry Beuvery et al. (1983). Carrier protein Tontini et al. (2016); Tontini et al. (2013)	Menactra (Sanofi Pasteur), MenQuadfi (Sanofi Pasteur), Menomune-A/C/Y/W-135 (Sanofi Pasteur), MENVEO (GlaxoSmithKline Biologicals SA)
*Neisseria meningitidis* serogroup C CPS	→9)-α-D-Neu*p*5Ac (7/8OAc)-(2→	Saccharide to protein ratio Micoli et al. (2020). Conjugation chemistry Carmenate et al. (2004). Carrier protein Baraldo et al. (2004); Tontini et al. (2016); Tontini et al. (2013)	Menactra (Sanofi Pasteur), MenQuadfi (Sanofi Pasteur), Menomune-A/C/Y/W-135 (Sanofi Pasteur), MENVEO (GlaxoSmithKline Biologicals SA)
*Neisseria meningitidis* serogroup X CPS	→1)-α-D-Glc*p*NAc4P-(4→	Carrier protein Tontini et al. (2016)	
*Neisseria meningitidis* serogroup W135 CPS	→6)-α-D-Gal*p*-(1→4)-α-D-Neu*p*5Ac (7/9OAc)-(2→	Carrier protein Tontini et al. (2016); Tontini et al. (2013)	Menactra (Sanofi Pasteur), MenQuadfi (Sanofi Pasteur), Menomune-A/C/Y/W-135 (Sanofi Pasteur), MENVEO (GlaxoSmithKline Biologicals SA)
*Neisseria meningitidis* serogroup Y CPS	→6)-α-D-Glc*p*-(1→4)-α-D-Neu*p*5Ac (7/9OAc)-(2→	Carrier protein Tontini et al. (2016); Tontini et al. (2013)	Menactra (Sanofi Pasteur), MenQuadfi (Sanofi Pasteur), Menomune-A/C/Y/W-135 (Sanofi Pasteur), MENVEO (GlaxoSmithKline Biologicals SA)
*Salmonella enteritidis* O-SP	→2)-[α-D-Tyv-(1→3)]-β-L-Man*p*-(1→4)-α-L-Rha*p*-(1→3)-[α-D-Glc*p*-(1→4)]_n_-α-D-Gal*p*-(1→	Conjugation chemistry Simon et al. (2011). Carrier protein Simon et al. (2011)	
*Salmonella typhi* Vi CPS	→4)-*α*-D-Gal*p*NAcA (3OAc)-(1→	Saccharide to protein ratio Micoli et al. (2020); An et al. (2011); Rondini et al. (2011). Saccharide length and saccharide to protein ratio Arcuri et al. (2017). Conjugation chemistry Kossaczka et al. (1999); Arcuri et al. (2017). Carrier protein Micoli et al. (2011); Cui et al. (2010); Arcuri et al. (2017).	
*Salmonella typhimurium* O-SP	→2)-[α-D-Par-(1→3)]-α-L-Man*p*-(1→4)-α-L-Rha*p*-(1→3)-[α-D-Glc*p*-(1→6)]-α-D-Gal*p*-(1→	Saccharide length and saccharide to protein ratio Svenson and Lindberg (1981). Saccharide to protein ratio Stefanetti et al. (2015). Conjugation chemistry Watson et al. (1992); Stefanetti et al. (2014); Stefanetti et al. (2015). Carrier protein Alexander et al. (2000).	
*Shigella dysenteriae* type 1 O-SP	→3)-α-L-Rha*p*-(1→2)-α-D-Gal*p*-(1→3)-α-D-Glc*p*NAc-(1→3)-α-L-Rha*p*-(1→	Saccharide to protein ratio Phalipon et al. (2009); van der Put et al. (2016). Saccharide length and saccharide to protein ratio Pozsgay et al. (1999)	
*Shigella flexneri* type 2a O-SP	→2)-α-L-Rha*p*-(1→2)-α-L-Rha*p*-(1→3)-α-L-Rha*p*-(1→3)-[α-D-Glc*p* (1→4)]-β-D-GlcpNAc-(1→	Saccharide to protein ratio van der Put et al. (2016). Carrier protein Passwell et al. (2001)	
*Shigella sonnei O-SP*	→4)-L-AltpNAcA-(1→3)-β-Fuc*p*NAc4N-(1→	Carrier protein Passwell et al. (2001); Pavliakova et al. (1999)	
*Staphylococcus aureus* type 8	→3)-β-D-Man*p*NAcA (4OAc)-(1→4)-α-L-Fuc*p*NAc-(1→3)-α-D-Fuc*p*NAc-(1→	Conjugation chemistry Fattom et al. (1992);Fattom et al. (1995). Carrier protein Fattom et al. (1995)	
*Streptococcus pneumoniae* type 1 CPS	→3)-D-AAT-α-Gal*p*-(1→4)-α-D-Gal*p*A (2/3OAc)-(1→3)-α-D-Gal*p*A-(1→		Prevnar 13 (Wyeth Pharmaceuticals), Prevnar 20 (Wyeth Pharmaceuticals), VAXNEUVANCE (Merck Sharp & Dohme)
*Streptococcus pneumoniae* type 3 CPS	→3)-*β*-D-GlcA-(1→4)-*β*-D-Glc*p*-(1→	Saccharide length and saccharide to protein ratio Benaissa-Trouw et al. (2001); Laferrière et al. (1997). Carrier protein Beuvery et al. (1982). Carrier protein Sigurdardottir et al. (2002)	Prevnar 13 (Wyeth Pharmaceuticals), Prevnar 20 (Wyeth Pharmaceuticals), VAXNEUVANCE (Merck Sharp & Dohme)
*Streptococcus pneumoniae* type 4 CPS	→3)-*β*-D-Man*p*NAc-(1→3)-*α*-L-Fuc*p*NAc-(1→3)-*α*-D-Gal*p*NAc-(1→4)-*α*-D-Gal*p*2,3(S)Py-(1→	Saccharide length and saccharide to protein ratio Peeters et al. (1991); Mawas et al. (2002). Carrier protein Sigurdardottir et al. (2002)	Prevnar 13 (Wyeth Pharmaceuticals), Prevnar 20 (Wyeth Pharmaceuticals), VAXNEUVANCE (Merck Sharp & Dohme)
*Streptococcus pneumoniae* type 5 CPS	→4)-β-D-Glc*p*-(1→4)-[α-L-Pne*p*NAc-(1→2)-β-D-Glc*p*A-(1→3)]-α-L-Fuc*p*NAc-(1→3)-β-D-Sug*p*-(1→		Prevnar 13 (Wyeth Pharmaceuticals), Prevnar 20 (Wyeth Pharmaceuticals), VAXNEUVANCE (Merck Sharp & Dohme)
*Streptococcus pneumoniae* type 6A CPS	→2)-*α*-D-Gal*p*-(1→3)-*α*-D-Glc*p*-(1→3)-*α*-L-Rha*p*-(1→3)-D-Rib-ol-(5→OPO_3_→	Carrier protein Chu et al. (1983).	Prevnar 13 (Wyeth Pharmaceuticals), Prevnar 20 (Wyeth Pharmaceuticals), VAXNEUVANCE (Merck Sharp & Dohme)
*Streptococcus pneumoniae* type 6B CPS	→2)-*α*-D-Gal*p*-(1→3)-*α*-D-Glc*p*-(1→3)-*α*-L-Rha*p*-(1→4)-D-Rib-ol-(5→OPO_3_→	Conjugation chemistry Steinhoff et al. (1994). Carrier protein (Dagan et al. (1997); Michon et al. (1998). Carrier protein Sigurdardottir et al. (2002)	Prevnar 13 (Wyeth Pharmaceuticals), Prevnar 20 (Wyeth Pharmaceuticals), VAXNEUVANCE (Merck Sharp & Dohme)
*Streptococcus pneumoniae* type 7F CPS	→4)-β-D-Glc*p*-(1→3)-[α-D-Glc*p*NAc-(1→2)-α-L-Rha*p*-(1→4)]-β-D-Gal*p*NAc-(1→6)-[β-D-Gal*p*-(1→2)]-α-D-Gal*p*-(1→3)-β-L-Rha*p* (2OAc)-(1→		Prevnar 13 (Wyeth Pharmaceuticals), Prevnar 20 (Wyeth Pharmaceuticals), VAXNEUVANCE (Merck Sharp & Dohme)
*Streptococcus pneumoniae* type 8 CPS	→4)-β-D-Glc*p*A-(1→4)-β-D-Glc*p*-(1→4)-α- D-Glc*p*-(1→4)-α-D-Gal*p*-(1→		Prevnar 20 (Wyeth Pharmaceuticals), Prevnar 20 (Wyeth Pharmaceuticals)
*Streptococcus pneumoniae* type 9V CPS	→4)-α-D-Glc*p*A (2/3OAc)-(1→3)-α-D-Gal*p*-(1→3)-β-D-Man*p*NAc	Carrier protein Sigurdardottir et al. (2002)	Prevnar 13 (Wyeth Pharmaceuticals), Prevnar 20 (Wyeth Pharmaceuticals), VAXNEUVANCE (Merck Sharp & Dohme)
	(4/6OAc)-(1→4)-β-D-Glc*p*-(1→4)-α-D-Glc*p*-(1→		
*Streptococcus pneumoniae* type 10A CPS	→5)-β-D-Gal*f*-(1→3)-β-D-Gal*p*-(1→4)-[β-D-Gal*f* (1→3)]-[β-D-Gal*p* (1→6)]-β-D-Gal*p*NAc-(1→3)-α-D-Gal*p*-(1→2)-D-Rib-ol-(5→ OPO_3_→		Prevnar 20 (Wyeth Pharmaceuticals)
*Streptococcus pneumoniae* type 11A CPS	→6)-[Gro (1→OPO_3_→4)]-α-D-Glc*p* (2/3OAc)-(1→4)-α-D-Gal*p*-(1→3)-β-D-Gal*p* (4/6OAc)-(1→4)-β-D-Glc_p_-(1→		Prevnar 20 (Wyeth Pharmaceuticals)
*Streptococcus pneumoniae* type 12F CPS	→4)-[α-D-Gal*p* (1→3)]-α-L-Fuc*p*NAc-(1→3)-β-D-Gal*p*NAc-(1→4)-[α-D-Glc*p-*(1→2)-α-D-Glc*p* (1→3)]-β-D-Man*p*NAcA-(1→		Prevnar 20 (Wyeth Pharmaceuticals)
*Streptococcus pneumoniae* type 14 CPS	→4)-*β*-D-Glc*p*-(1→6)-[*β*-D-Gal*p*-(1→4)]-*β*-D-Glc*p*NAc-(1→3)-*β*-D-Gal*p*-(1→	Saccharide to protein ratio Polonskaya et al. (2017). Conjugation chemistry Mawas et al. (2002). Carrier protein van de Wijgert et al. (1991). Carrier protein Dagan et al. (1997); Michon et al. (1998); Sigurdardottir et al. (2002)	Prevnar 13 (Wyeth Pharmaceuticals), Prevnar 20 (Wyeth Pharmaceuticals), VAXNEUVANCE (Merck Sharp & Dohme)
*Streptococcus pneumoniae* type 15B CPS	→6)[-α-D-Gal*p* (2/3/4/6 OAc)-(1→2)-[Gro-(2→OPO_3_→3)]-β-D-Gal*p*-(1→4)]-β-D-GlcpNAc-(1→3)-β-D-Gal*p*-(1→4)-β-D-Glc*p*-(1→		Prevnar 20 (Wyeth Pharmaceuticals)
*Streptococcus pneumoniae* type 18C CPS	→4)-*β*-D-Glc*p*-(1→4)-[*α*-D-Glc*p* (6OAc) (1→2)][Gro-(1→OPO_3_→3)]-*β*-D-Gal*p*-(1→4)-*α*-D-Glc*p*-(1→3)-*β*-L-Rha*p*-(1→	Carrier protein Sigurdardottir et al. (2002)	Prevnar 13 (Wyeth Pharmaceuticals), Prevnar 20 (Wyeth Pharmaceuticals), VAXNEUVANCE (Merck Sharp & Dohme)
*Streptococcus pneumoniae* type 19A CPS	→4)-β-D-Man*p*NAc-(1→4)-α-D-Glc*p*-(1→3)-α-L-Rha*p*-(1→OPO_3_→		Prevnar 13 (Wyeth Pharmaceuticals), Prevnar 20 (Wyeth Pharmaceuticals), VAXNEUVANCE (Merck Sharp & Dohme)
*Streptococcus pneumoniae* type 19F CPS	→4)-*β*-D-Man*p*NAc-(1→4)-*α*-D-Glc*p*-(1→2)-*α*-L-Rha*p*-(1→OPO_3_→	Carrier protein Dagan et al. (1997); Michon et al. (1998); Sigurdardottir et al. (2002)	Prevnar 13 (Wyeth Pharmaceuticals), Prevnar 20 (Wyeth Pharmaceuticals), VAXNEUVANCE (Merck Sharp & Dohme)
*Streptococcus pneumoniae* type 22F CPS	→4)-*β*-D-Glc*p*A-(1→4)-[*α*-D-Glc*p*-(1→3)]-*β*-L-Rha*p* (2OAc)-(1→4)-*α*-D-Gal*p*-(1→3)-*α*-D-Gal*f*-(1→2)-*α*-L-Rha*p*-(1→		Prevnar 20 (Wyeth Pharmaceuticals), VAXNEUVANCE (Merck Sharp & Dohme)
*Streptococcus pneumoniae* type 23F CPS	→4)-*β*-D-Glc*p*-(1→4)-[*α*-L-Rha*p*-(1→2)]-[Gro-(2→OPO_3_→3)]-*β*-D-Gal*p*-(1→4)-*β*-L-Rha*p*-(1→	Conjugation chemistry Steinhoff et al. (1994). Carrier protein Dagan et al. (1997); Michon et al. (1998); Sigurdardottir et al. (2002)	Prevnar 13 (Wyeth Pharmaceuticals), Prevnar 20 (Wyeth Pharmaceuticals), VAXNEUVANCE (Merck Sharp & Dohme)
*Streptococcus pneumoniae* type 33F CPS	→3)-*β*-D-Gal*p*-(1→3)-[*α*-D-Gal*p*-(1→2)]-*α*-D-Gal*p*-(1→3)-*β*-D-Gal*f* (OAc)-(1→3)-*α*-D-Glc*p*-(1→5)-*β*-D-Gal*f*-(1→		Prevnar 20 (Wyeth Pharmaceuticals), VAXNEUVANCE (Merck Sharp & Dohme)
*Vibrio cholerae* O1 O-SP	→2)-α-D-Per*p*N-(3-deoxy-L-glycero-tetronyl)-(1→	Conjugation chemistry Gupta et al. (1992); Saksena et al. (2006)	

*This table was adopted from the complete list of ‘Vaccines Licensed for Use in the United States’ provided by the US Food and Drug Administration website (https://www.fda.gov/). Content current as of: April 18, 2023.

Conjugation of a saccharide to a suitable carrier protein converts it into a T-dependent antigen (Mäkelä and Käyhty, 2002; Weintraub, 2003) with B and T cells now able to interact in the generation of the antibody response (Mitchison, 1971a; b;^2004^). Importantly, conjugate vaccines can induce strong immune responses from birth (Lindberg, 1999; Pozsgay, 2000; Ravenscroft and Jones, 2000; Lesinski and Westerink, 2001; Mäkelä and Käyhty, 2002; Jones, 2005; Lucas et al., 2005). The PS component of the vaccine binds to surface immunoglobulin of PS-specific B cells. Following internalization of the vaccine, the protein component is processed and resulting peptide fragments are presented to the T cell receptor of CD4^+^ peptide-specific T cells in the peptide-binding groove of major histocompatibility complex class II molecules (MHCII). In addition to this cognate interaction, further signals are essential in eliciting CD4^+^ T cell help for the B cell (Borriello et al., 1997; Guttormsen et al., 1999). An alternative mechanism for the activation of the immune response by glycoconjugate vaccines has been also proposed, with the carbohydrate fragments of glycopeptides, produced by glycoconjugate vaccine processing within antigen-presenting cells, being recognized by CD4^+^ T cells while the peptide moieties allow binding to MHCII molecules (Lai and Schreiber, 2009; Avci et al., 2011). In this regard, a recent study demonstrated that the mechanisms of antigen presentation to T cells may also depend on the structure of the carbohydrate itself, and peptide or glycopeptide presentation to T cells could be of varying importance (Sun et al., 2019).

When B cells receive T cell help, they proliferate and differentiate into plasma cells, with class switching, particularly to IgG, and memory B cells. Memory B cells can rapidly proliferate and differentiate into plasma cells on subsequent encounter with the specific antigen producing high antibody titers (Insel and Anderson, 1986; Guttormsen et al., 1998; Tangye et al., 2003; Guirola et al., 2006; Pichichero, 2009). Antibody avidity is increased through affinity maturation in germinal centers (Goldblatt et al., 1998). Conjugate vaccines are normally not associated with hyporesponsiveness (Poolman and Borrow, 2011).

Persistence of antibodies following immunization, immunological memory and herd immunity (John and Samuel, 2000; Ramsay et al., 2003; Lexau et al., 2005) are important factors for long-term protection against invasive diseases from encapsulated bacteria (Pollard et al., 2009; Trück and Pollard, 2010; Blanchard-Rohner and Pollard, 2011). Despite induction of immunological memory, serum antibody levels and vaccine effectiveness can wane following infant immunization (MacLennan et al., 2000; Rennels et al., 2001; Borrow et al., 2002; Dagan et al., 2004; Trotter et al., 2004; Snape et al., 2005; Auckland et al., 2006; Tsai et al., 2006; Blanchard Rohner et al., 2008; Perrett et al., 2010). Booster doses of vaccine can maintain serum antibody levels above the protective threshold (Clutterbuck et al., 2006). Strategies to enhance the initial B cell response to immunization, using adjuvants and adjusting immunization schedules, can improve antibody persistence, although this needs further investigation (Borrow et al., 2003; Kelly et al., 2006; Blanchard Rohner et al., 2008; Clutterbuck et al., 2008; Kelly et al., 2009; Rappuoli, 2018).

## 2 Traditional and newer technologies for glycoconjugates

**FIGURE 1:**
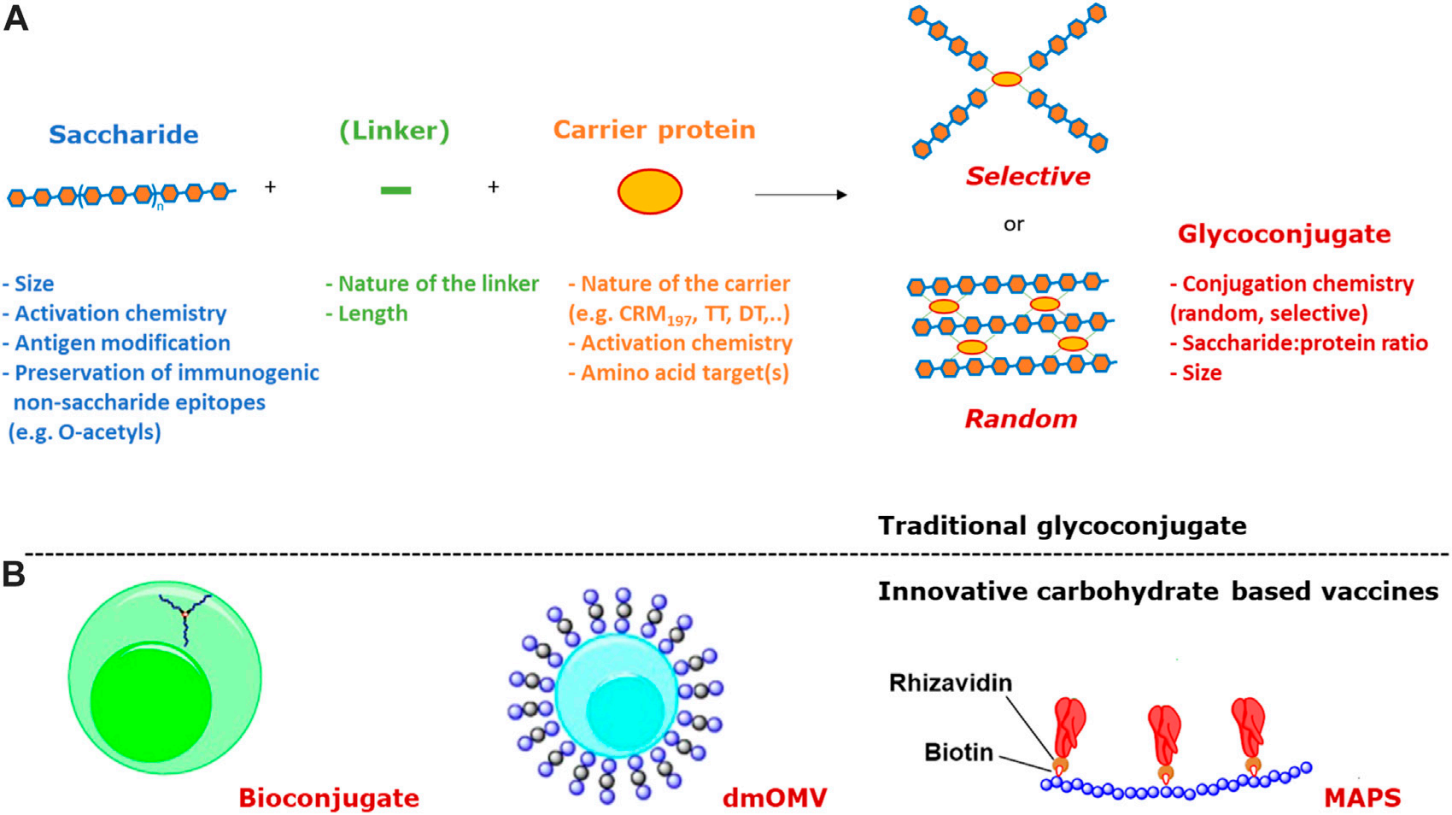
**(A)** The two main approaches to traditional conjugation of saccharides to proteins (linkers are not always present) and main variables impacting immunogenicity; **(B)** Innovative carbohydrate based vaccines based on bioconjugation, dmOMV and MAPS technologies.

**FIGURE 2:**
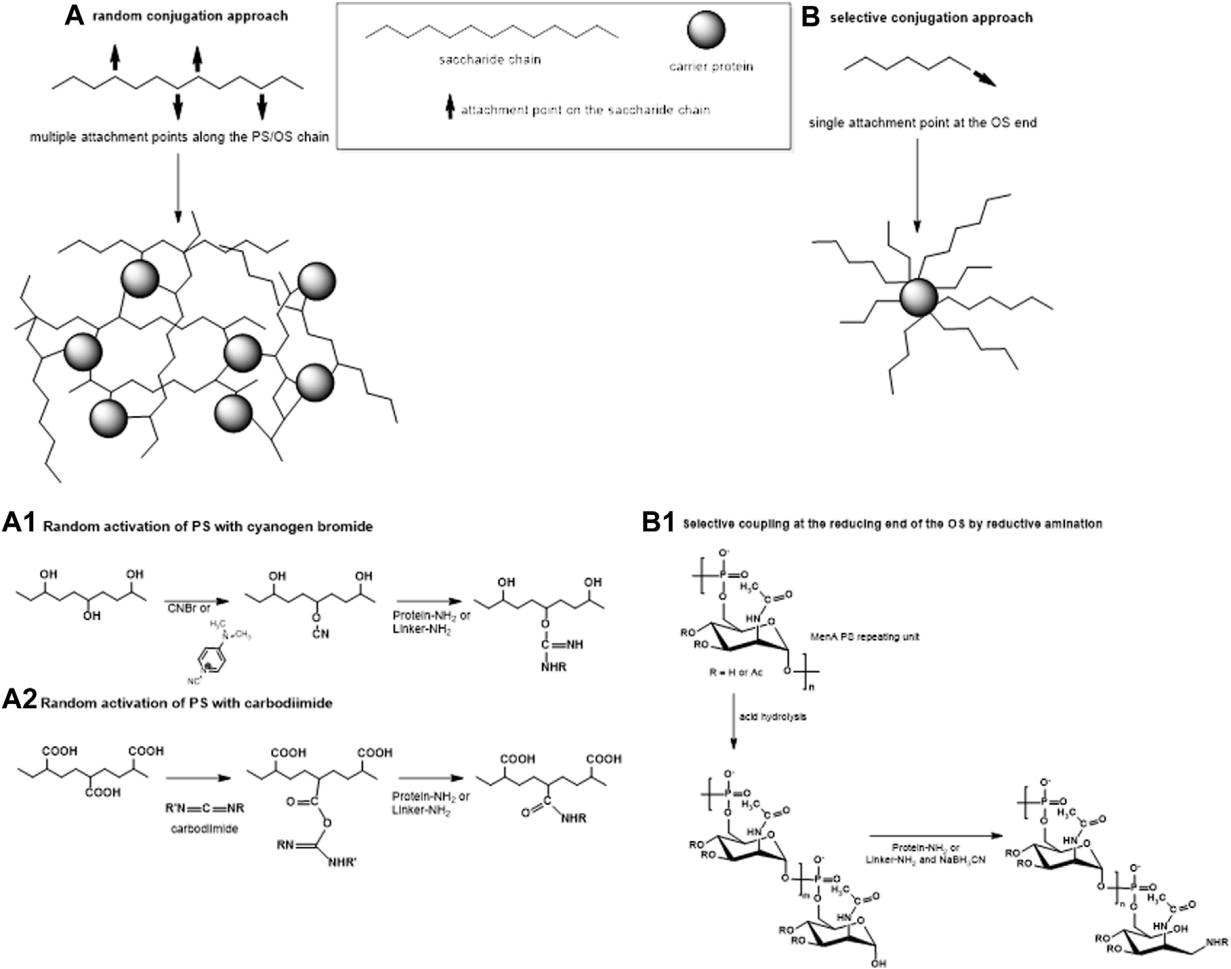
Two main approaches of conjugation: **(A)** random linkage sites along the PS or OS chain, resulting in high molecular weight cross-linked and rather undefined and heterogeneous conjugate structures and **(B)** selective attachment at the terminal end of the OS, resulting in better-defined conjugates without chemical modification of the saccharide chain. **(A1–A2)** Some commonly used methods for random activation of the PS using OH groups (1) or COOH groups (2) along the saccharide chain. **(B1)** An example of selective conjugation after depolymerization of the PS by mild acid hydrolysis. Mild hydrolysis, for example, of MenA PS, generates one terminal aldehyde group per shorter saccharide chain. Resulting aldehyde groups are linked to the protein or to the spacer by reductive amination.

Traditionally, the synthesis of glycoconjugate vaccines requires a covalent linkage between the saccharide and the carrier protein. Many different conjugation chemistries have been proposed (Pozsgay, 2000; Frasch, 2009; Costantino et al., 2011; Micoli et al., 2019), but all follow two main approaches: 1) random linkage along the PS or OS chain (Figures 1, 2A); 2) selective attachment at the terminal end of OS (Figures 1, 2B). The random chemistry approach results in high molecular weight (MW), and cross-linked and rather undefined heterogeneous structures, while the selective approach produces better-defined structures, avoiding chemical modification of the saccharide chain (Pozsgay, 2000; Wang et al., 2003; Jones, 2005; Lucas et al., 2005). The possibility to conjugate chemically the PS at specific sites on the carrier protein has also been explored. These approaches facilitate structure-activity relationship investigation and preservation of key protein epitopes allowing a dual role of the protein component as carrier and antigen. Spacer molecules are often introduced between the saccharide and protein to reduce steric hindrance and facilitate interaction between both moieties (Kubler-Kielb and Pozsgay, 2005; Lees et al., 2006; Floyd et al., 2009; Costantino et al., 2011).

An innovative methodology, known as bioconjugation (Figure 1B), has been developed for *in vivo* production of glycoproteins, where both the saccharide antigen and the carrier protein are expressed in *Escherichia coli* cells and coupled *in vivo* (Wacker et al., 2002). Glycoconjugate production in *E. coli* requires the presence of genome clusters encoding the bacterial polysaccharide, a plasmid encoding the carrier protein, and the oligosaccharyl transferase (OTase; traditionally the enzyme PglB from *C. jejuni*). The lipid undecaprenyl pyrophosphate (Und-PP), present in the cytoplasmic membrane of *E. coli*, works as an anchor for the construction of oligo- and polysaccharides. Then, the glycan moiety is flipped in the periplasmatic space and elongated by a polymerase. The PglB transfers Und-PP-linked glycans to the appropriate consensus acceptor sequence (i.e., D/E-X1-N-X2-S/T, where X1 and X2 can be any amino acid except proline) inserted in the carrier protein. This allows for precise control of where saccharide antigens are attached to the protein.

Alternative carrier systems, such as Outer Membrane Vesicles (OMV) have also been explored, which can facilitate antigen uptake by APCs, ensure display of the carbohydrate in multiple copies, and confer self-adjuvanticity through the presence of Toll-like receptor stimulatory molecules in the case of OMV (Berti and Micoli, 2020). Mutant-derived OMV (mdOMV), also called GMMA (Generalized Modules for Membrane Antigen) are OMV produced by Gram-negative bacteria which have been genetically manipulated to increase OMV release and reduce reactogenicity of lipid A. mdOMV (Figure 1B) have been proposed as delivery vehicles for O-antigens naturally present on their surface, or carrier for chemical conjugation of heterologous polysaccharides (Micoli and MacLennan, 2020). Also *E. coli* strains not expressing the O-SP portion of LPS molecules have been genetically manipulated for the biosynthesis and surface expression of heterologous polysaccharides anchored to lipid A-core as acceptors (Valguarnera and Feldman, 2017).

Covalent linkage of PS to the carrier protein has been thought fundamental to the immunological properties of conjugate vaccines. However, alternative approaches, such as the affinity-based coupling approach of the Multiple antigen-presenting system (MAPS) technology, have tested the co-delivery of the two components through non-conventional biotin-rhizavadin connection (Zhang et al., 2013) (Figure 1B).

The physical characteristics of traditional and innovative glycoconjugate vaccines (Figure 1), including saccharide length, saccharide to protein ratio, carrier protein, conjugation method and linker, can affect the magnitude, quality and persistence of the antibody response elicited, as well as cellular responses. Thus, these variables can have a marked influence on the success of a vaccine and a better understanding of the influence of these parameters can facilitate the rational design of glycoconjugate vaccines with improved immunogenicity and enhanced ability to protect. Here, we have conducted a comprehensive review of studies that compare the immunogenicity of conjugate vaccines differing in such characteristics. Table 1 reports the structure of the polysaccharides for which the impact of certain variables on the immunogenicity of corresponding glycoconjugates has been investigated. Saccharides used in licensed vaccines are highlighted.

## 3 Saccharide length and saccharide to protein ratio

We have recently reviewed the impact that PS chain length can have on the immune response elicited by glycoconjugate vaccines (Stefanetti et al., 2022). Saccharides can be isolated from bacterial cultures and are often reduced in size through different methodologies in order to simplify manufacture; improve conjugation yields, purification and consistency; and facilitate conjugate characterization. Shorter OS can be produced by organic synthesis and/or enzymatic approaches as well. According to the length of the conjugated sugars, different optimal saccharide to protein ratios can be identified, these two parameters appearing to be strongly interconnected. A high glycan loading could be needed for conjugation of short OS to result in an optimal immune response, while a lower density could be sufficient for longer PS. More recently nanoparticle carrier systems have been explored for the display of carbohydrates, offering multimeric presentation of glycan epitopes coupled with special chemico-physical properties of nano-sized particles. Control of saccharide loading on the protein can be critical when the protein plays a double role of carrier and antigen and, despite conjugation, the antigenicity of the protein needs to be preserved. Below, examples of saccharide loading impacting the immune response elicited by saccharide of defined chain length are reported.

### 3.1 *Haemophilus influenzae* type b

Anderson et al. (1989), studying Hib OS directly coupled at one end to CRM_197_ in human adults and 1-year-old infants, found that hapten loading (in a range of 0.02–0.1 μg ribose/μg protein) was a more critical variable than OS length, in the range of 4–12 repeating units. Increased saccharide loading produced enhanced antibody response.

### 3.2 Meningococcus

OMVs combine antigen presentation with the immunopotentiator effect of the Toll-like receptor agonists naturally present on these nanoparticles. When different average numbers of MenA or MenC OS (average size of 4.5 kDa) were conjugated to OMV membrane proteins, no major impact of OS density (in the range of 650–4,500 sugar chains per OMV) was observed on anti-PS IgG response and functionality of antibodies elicited in mice. However, the control of glycosylation density could be useful to fully preserve the immune response induced by protein components of the OMV (Micoli et al., 2020).

### 3.3 *Salmonella*

An et al. (2011) studied impact of full-length Vi loading on a traditional carrier protein like Diphtheria Toxoid (DT). *C*onjugates produced using random chemistry with ADH linker were tested in mice. The anti-Vi antibody response progressively increased with increasing DT to PS content. Rondini et al. (2011), using a similar conjugation approach as An et al., but with CRM_197_ as carrier, found that a weight to weight ratio of full length Vi to CRM_197_ of 0.9 and 2.1 produced more antibodies than a ratio of 10. Recently the impact of saccharide to protein ratio on the immunogenicity of full length (165 kDa) and fragmented Vi (43 kDa) randomly conjugated to CRM_197_ was evaluated, albeit over a limited range of ratios. Altering the ratio of Vi to protein from 0.8 to 1.4 (for full length Vi) and from 0.26 to 0.85 (for fragmented Vi) did not influence the anti-Vi IgG response in mice (Arcuri et al., 2017).

Conjugates differing for number of PS chains to OMV were produced by conjugation of *S.* Typhi Vi PS of 48.5 kDa (93 or 17 chains per OMV) or 3.8 kDa (138 or 55 chains per OMV) to *S.* Typhimurium OMV. No impact of antigen density on Vi-specific serum IgG response in mice was observed (Micoli et al., 2020).

Svenson and Lindberg (1981) found that immunogenicity of *Salmonella* Typhimurium octasaccharide-BSA conjugates in mice was enhanced by increasing the molar ratio of saccharide to protein from 7 to 23. However, with a longer *Salmonella* Typhimurium O-SP, composed of approximately 25 repeating units, robust immunogenicity was obtained even with a single attachment site, depending on the targeted amino acid on CRM_197_ (Stefanetti et al., 2015). Indeed the conjugate elicited similar bactericidal antibody response compared to a random conjugate with multiple saccharide chains in mice when the O-SP chain was linked to one of the disulfide bonds of CRM_197_, but not to specific lysine residues.

### 3.4 Shigella

For *Shigella flexneri* 2a conjugates with synthetic pentadecasaccharide, an average molar ratio of two saccharide chains per protein induced no anti-LPS antibodies in mice, and an average ratio of 14 was more immunogenic than eight (Phalipon et al., 2009). More recently (van der Put et al., 2016), comparison of four *S. flexneri* 2a glycoconjugates only differing by their pentadecasaccharide to Tetanus Toxoid (TT) ratio confirmed that hapten loading is critical for immunogenicity with an optimal saccharide to protein ratio in the range of 17 ± 5, supporting the selection of a synthetic conjugate with average loading of 15 OS per TT which was subsequently tested in a Phase 1 clinical study (Cohen et al., 2021). Pozsgay et al. (1999), testing immunogenicity of *Shigella dysenteriae* type 1 conjugates in mice, found that the optimal carbohydrate chain density differed with OS length, highlighting the interrelatedness of these parameters.

### 3.5 Streptococcus pneumoniae

Benaissa-Trouw et al., 2001) found that immunogenicity in mice of synthetic di-, tri-, and tetrasaccharides of *S. pneumoniae* serotype (PnPS) 3 conjugated to CRM_197_ was not influenced by the carbohydrate to protein ratio over a molar range of 2.9–12. Similar results were obtained in rabbits by Laferrière et al. (1997), who compared PnPS 3 of 8 repeating units differing for saccharide to protein molar ratio of 6.3, 8.1, and 12.5, and PnPS 19F OS of 17 repeats with 2.8 or 5.5 chains terminally linked to TT. However, the majority of studies have found that saccharide to protein ratio does affect immunogenicity of glycoconjugates.

Peeters et al. (1991) showed that PnPS 4 (4–120 kDa)-TT conjugates in the range of 0.3–2.8 saccharide to protein molar ratio were more immunogenic in mice than conjugates with a lower saccharide loading, while the immunogenicity of OS (12 repeating units, 9.6 kDa)-TT conjugates increased with higher saccharide density (PnPS:TT molar ratio 50 > 25 > 14). Also, for PnPS 14 synthetic tetrasaccharides coupled to CRM_197_, over the molar ratio ranges of 5–24, the highest OS loading corresponded to the highest level of antibodies in mice (Mawas et al., 2002).

Controlled reproducible loading of approximately 20–200 synthetic tetrasaccharides of PnPS 14 was achieved with nanoparticles made of 180 repetitive units assembled in a virus-like particle (VLP) (Polonskaya et al., 2017). Mice were immunized with conjugates bearing on average 20, 40, 80, or 200 OS per particle. No detectable anti-glycan antibody response was produced in mice immunized with VLPs containing an average of 20 OS, and a certain variability in both the primary and the secondary antibody responses was obtained with VLPs with an average of 40 OS. In contrast, conjugates with higher glycan densities produced similarly potent antibody responses in animals.

## 4 Conjugation chemistry

Conjugation chemistry plays an important role in determining the immunogenicity of glycoconjugate vaccines. Different approaches can be used to covalently attach the carbohydrate antigen to a carrier protein, and both the efficiency of the chemistry chosen, and the availability of reacting sites in the two molecules can influence critical immunological variables such as sugar to protein ratio and molecular size of the resulting vaccine (Jones, 2005; Costantino et al., 2011; Berti and Adamo, 2018). In addition, the use of linkers between the saccharide antigen and the carrier protein can facilitate the conjugation between those high-molecular weight biomolecules and can also affect the immunogenicity of these vaccines.

The method of carbohydrate coupling to the protein depends on the saccharide structure. Hydroxyl groups along the saccharide can be randomly activated by cyanylation (Schneerson et al., 1980; Chu et al., 1983; Lees et al., 1996; Shafer et al., 2000) (Figure 2A1). Sodium periodate can be used to oxidize two adjacent carbons with hydroxyl groups generating two aldehyde groups and breaking the C–C bond in the process. For some saccharides, like Hib and meningococcal serogroup C (MenC) PS, this produces shorter saccharides with reactive terminal aldehyde groups (Anderson et al., 1986), while for other saccharides, like MenA PS (Lee et al., 2009), this opens the sugar ring, without PS fragmentation. In both cases, the resulting carbonyl groups can be used for reductive amination with the protein directly or with a linker. Some PS, like Hib and meningococcal PS, can be fragmented by mild acid hydrolysis while maintaining the repeating unit motif, generating an aldehyde group at the reducing end only which is then available for conjugation (Costantino et al., 1992; Costantino et al., 1999; Ravenscroft et al., 1999; Matjila et al., 2004) (Figure 2B1). *Salmonella* Typhi and *Staphylococcus aureus* PS are resistant to acid hydrolysis and their conjugation has been achieved by activating carboxyl groups with carbodiimide (Szu et al., 1987; Fattom et al., 1995; Kossaczka et al., 1997) (Figure 2A2).

Amino groups on lysine residues or carboxyl groups on aspartic or glutamic acid residues are the main linkage points used on proteins for conjugation, sometimes after modification with linkers exposing chemical groups for conjugation. Recent years have seen the rise of novel site-selective protein glycosylation methods aiming to provide more homogeneous and well-defined glycoconjugate constructs, facilitating both conjugate physico-chemical characterization and the investigation of structure-activity relationship. Chemical, by targeting cysteine or tyrosine residues, or enzymatic, by modifying glutamine, glycine or lysine residues, approaches have been explored. Recombinant proteins have also been engineered by inserting unnatural amino acids, non-proteinogenic amino acids that either occur naturally or are chemically synthesized, in specific regions of the protein sequence (Hu et al., 2016). This strategy can enhance the functionality of proteins or peptides by introducing specific chemical groups or reactive moieties at specific sites along the protein sequence.

Significant progress has been made in recent years in identifying enzymes involved in natural glycan conjugation in bacteria, creating new opportunities for glycoconjugate vaccine production (Terra et al., 2012; Cuccui and Wren, 2015; Moeller et al., 2021). The oligosaccharyltransferase (OST) enzyme PglB from *Campylobacter jejuni* has been used to connect bacterial PS to carrier proteins through both chemoenzymatic and *in vivo* methods, providing an effective way to directly link sugars to a specific amino acid sequence of the protein. Both N-linked and O-linked glycosylation techniques have been employed, and ongoing efforts aim to expand these capabilities.

The use of peptides as carriers instead of proteins can result in conjugates where multiple peptides are attached only in one position (generally the C- or N-terminus) to multiple positions along the saccharide chain (Alexander et al., 2000; Alexander et al., 2004; Ingale et al., 2007; Xin et al., 2008). The recent finding that glycopeptide fragments may be involved in the immunological synapse and presented to TCR by MHC-II molecules (Avci et al., 2011) has focused attention on the fine details of the glycan-protein linkage, in particular the specific amino acid residue(s) involved. Targeting different amino acids for conjugation results in the formation of distinct glycopeptides with potentially different abilities to raise a specific T cell-dependent immune response against the carbohydrate portion. Interestingly, the need for covalent conjugation has been challenged recently by new technologies. These include the Protein Capsular Matrix Vaccine (PCMV; Matrivax) platform which enables the capture of both the sugar antigen and the carrier protein in a polymer matrix (Thanawastien et al., 2015), and the ‘MAPS’ (Multiple Antigen Presenting System) technology, where covalent binding is substituted by affinity-based coupling of proteins engineered with rhizavidin moieties and biotynilated PS (Zhang et al., 2013).

### 4.1 Dextrans

Seppala et al. (Seppälä and Mäkelä, 1989) studied the effect of random and selective (with and without ADH linker) chemistries to conjugate dextrans to Chicken Serum Albumin (CSA). Selective chemistry using short saccharide molecules (4–10 kDa) induced much higher antibody responses than random activation, and linker presence did not affect immunogenicity. For 40 kDa dextrans, conjugates prepared with random chemistry were more immunogenic than with selective methods.

### 4.2 Escherichia coli

For the design of vaccines to prevent against enteritis caused by *Escherichia coli,* four *E. coli* O111 glycoconjugates were synthesized and compared in mice. The saccharide was generated through the processing of its LPS using either acetic acid (O-SP) or the organic base hydrazine (DeA-LPS).

The O-SP had a reduced concentration of colitose. The products (O-SP or DeA-LPS) underwent derivatization with ADH (random derivatization) or thiolation using N-succinimidyl-3 (2-pyridyldithio) propionate (SPDP) (selective derivation on the aminoethanol moiety of the core region). The resulting four derivatives were then covalently attached to TT. Immunization studies in mice revealed that, out of the four conjugates tested, DeA-LPS–TT random conjugate produced using ADH as linker induced the most significant amount of anti-LPS antibodies, despite all conjugates tested having similar sugar to protein ratio (0.7–1.4). The authors suggested that the superior immunogenicity of this glycoconjugate over the other three conjugates tested can be attributed to several factors, including the increased retention of colitose, higher molecular weight (O-SP molecular weight population possesses two peaks with Kds of 0.2 and 0.5, while DeA-LPS had three peaks with Kd < 0.1, 0.4 and 0.6), the attachment of DeA-LPS to TT at multiple points, and the physicochemical characteristics of ADH (Gupta et al., 1995).

### 4.3 Escherichia hermannii

*Escherichia hermannii* O-SP linked directly to BSA gave earlier and more persistent antibody response in mice than when ADH linker was used, both approaches using random conjugation (Jacques and Dubray, 1991). Specifically, the direct conjugation required a lower dosage to induce a similar IgG response, that was sustained for at least 4 months.

### 4.4 Group B *Streptococcus*

Shen et al. compared the direct conjugation of GBS type III capsular PS, activated by random periodate oxidation, to recombinant cholera toxin B subunit (*r*CTB) via reductive amination, with two other random conjugation chemistries that employed cystamine and SPDP, or ADH, respectively. They also evaluated the impact of glycoconjugate size on immunogenicity by separating the conjugates into large and small molecular weight batches before intranasal immunization in mice. Sugar to protein ratios were comparable among the constructs (respectively around 0.9 for large size conjugates and around 1.1–1.5 for small size conjugates). Results showed that the large and small conjugates made by reductive amination or via ADH linking induced high capsular PS-specific IgG levels in serum, lungs, and vagina, with the larger batches eliciting higher IgA titers. In contrast, the conjugates made via SPDP linking were less immunogenic. When comparing sizes, the immunogenicity of smaller-molecular weight conjugates was generally lower than that of the larger-molecular weight conjugates, as evidenced by a reduced anti-capsular PS IgA immune response. Notably, the larger-molecular weight conjugate made by direct conjugation via reductive amination elicited the highest anti-capsular PS IgA titers in both tissues and serum, indicating that the molecular size of the conjugates influences the immune response (Shen et al., 2001).

Wessels et al. conducted a study on GBS type III capsular PS-TT conjugates, which were obtained through the reductive amination of sodium periodate oxidized-capsular PS (Wessels et al., 1998). The researchers identified an optimal threshold for PS-protein cross-linking to induce high titers of functional antibodies in mice. To examine the effect of PS-protein cross-linking on immunogenicity, the researchers constructed a series of vaccine candidates using type III GBS PS with various proportions of sialic acid residues modified by periodate oxidation (18%, 35%, 66%, or 89%). All four conjugates had a comparable sugar to protein ratio (total saccharide content 42%–50%). Immunogenicity studies in mice showed a significant direct relationship between the extent of PS-protein cross-linking and PS-specific antibody response to the conjugate. III66%-TT elicited significantly greater opsonic killing activity in antiserum than III18%-TT, III35%-TT, or III89%-TT, suggesting that some antibodies evoked by highly cross-linked conjugates were directed to a nonprotective epitope. Indeed, some antibodies recognized a new epitope induced by extensive oxidation of the PS, but not present on the native PS and therefore unlikely to be relevant for protective immunity (Cox et al., 2017).

The impact of conjugating GBS type V capsular PS**,** again activated randomly by oxidation, to specific lysine or tyrosine residues of the GBS67 pilus protein, which serves both as protein carrier and antigen, was evaluated. Protein-selective conjugates, made by click chemistry, thiol-maleimide addition or microbial Transglutaminase (mTGase) catalyzed insertion, were compared to GBS type V conjugates created by attaching the PS to either GBS67 or CRM_197_ proteins by random chemistry. Immunological study in mice revealed that all the site-directed constructs effectively induced high levels of anti-PS and anti-protein IgG, resulting in osponophagocytic killing of bacterial strains expressing either PSV or GBS67. Among the different site-directed conjugates tested, the tyrosine-directed thiol-maleimide addition was found to be the most effective in generating functional IgG against both PS and protein (as evidenced by *in vitro* opsonophagocytic killing titers), comparable to controls prepared by random conjugation, without inducing anti-linker antibodies (Nilo et al., 2015b).

### 4.5 *Neisseria* meningitidis

With conjugates of MenA PS linked to TT by random chemistries, use of glycine or 6-amino-n-hexanoic acid spacers led to four-fold higher protein to PS ratios compared with no spacer (Beuvery et al., 1983). Conjugates with a spacer were more immunogenic after the second, but not the first immunization in mice. With MenC PS (60 kDa) coupled to the meningococcal P64k protein, a random conjugate obtained by direct reductive amination after saccharide oxidation with NaIO_4_ (protein to saccharide ratio of 1.43) elicited higher anti-PS IgG titers and higher bactericidal activity than when carbodiimide condensation following saccharide derivatization with ADH was used (protein to saccharide ratio of 0.14) (Carmenate et al., 2004). However, the two conjugates had very different sugar to protein ratio, which complicates the evaluation of the impact of the conjugation chemistry on the immunogenicity response. Some studies have reported induction of high levels of linker-specific antibodies (Peeters et al., 1991; Buskas et al., 2004). In the presence of a strongly immunogenic glycan such as MenC PS, the chemical link between ADH and the PS is immunologically silent, but becomes immunodominant in the presence of a poor immunogen such as MenB PS (Bartoloni et al., 1995).

### 4.6 *Salmonella*

For *Salmonella* Enteritidis O-SP, the direct conjugation to flagellin monomers by random activation (two conjugates tested with saccharide to protein ratios of 0.97 and 0.26) induced the same IgG levels and protection against subsequent challenge in mice as with selective aminooxyoxime thioether chemistry using diaminooxy cysteamine and *N*-(γ-maleimidobutyloxy)-sulfosuccinimide ester linkers (saccharide to protein ratio = 0.45) (Simon et al., 2011).

For *Salmonella* Typhi Vi conjugates, PS linked to recombinant *Pseudomonas aeruginosa* exotoxin A (*r*EPA) by random chemistry was more immunogenic using an ADH linker (saccharide to protein ratio of 1.12) than with cystamine and SPDP (saccharide to protein ratio of 1.06) in humans (Kossaczka et al., 1999). More recently, different chemistries have been tested to link Vi of 43 kDa to CRM_197_ carrier protein, using -Ethyl-3-(3-Dimethylaminopropyl)carbodiimide, Hydrochloride (EDAC) or click chemistry for random conjugates, and EDAC chemistry or thiol chemistry with linkers with different lengths for selective conjugates, targeting different amino acids (Asp/Glu or Lys). No significant differences were observed in the anti-Vi IgG response induced in mice between the difference glycoconjugate constructs (Arcuri et al., 2017).

Recently, a classical glycoconjugate was compared with a MAPS construct for a carbohydrate-based vaccine against *Salmonella* Typhi. To assess the efficacy of the different vaccines, adoptive transfer of B and T cells from immunized wild-type C57BL/6 mice to Rag−/− mice were conducted. Specifically, the recall of memory response for a classical Vi-DT conjugate and a *r*EPA-Vi MAPS complex was compared. The results showed that the anti-Vi IgG antibody titers induced by the two conjugate vaccines were comparable (Zhang et al., 2022).

Watson et al. (1992) found that *Salmonella* Typhimurium O-SP-TT conjugates, produced by random activation and ADH linker (saccharide to protein ratio of 0.6) were more immunogenic in mice than when selective chemistry with ADH (saccharide to protein weight ratio of 0.1) was used, starting with O-SP having a bimodal molecular weight population with a major peak at 30 kDa and a minor peak at 119 kDa. Immunogenicity was also positively affected by the amount of linker incorporated, which had a positive linear relationship with the sugar to protein ratio in the final conjugate. However, for O-SP-CT conjugates produced by random (saccharide to protein ratio of 1.06) compared with two conjugates made by selective activation (saccharide to protein ratio of 0.23 and 0.5), using SPDP linker, there was no difference in the elicited immunogenicity. Using CRM_197_ as carrier protein (Stefanetti et al., 2014), *Salmonella* Typhimurium O-SP **(**20.5 kDa**)** was randomly activated by oxidation, respectively using a mild oxidizing agent, TEMPO (two conjugates with saccharide to protein ratio of 0.56 and 0.38), or NaIO_4_ (saccharide to protein ratio of 0.72), which opens the sugar units impacting O-SP epitopes and conformation. Selective activation at the reducing end of the saccharide was obtained by using two different lengths of the spacer (NH_2_-SIDEA (saccharide to protein ratio of 1.42) and ADH-SIDEA (saccharide to protein ratio of 1.74)) between the carrier protein and the sugar reducing end. The random and selective conjugates induced similar anti-OAg IgG responses in mice; however, the random conjugates induced antibodies with higher bactericidal activity. Notably, the use of TEMPO oxidation resulted in antibodies with the highest bactericidal activity. Another study involved synthesis of glycoconjugates with defined connectivity by targeting different amino acids on CRM_197_, including two novel methods for site-selective glycan conjugation (Stefanetti et al., 2015). Conjugation at the cysteine residues C186-201 bond resulted in higher anti O-SP bactericidal antibodies than coupling to lysines K37/39.

### 4.7 Staphylococcus aureus

Similar to *Salmonella* Typhi Vi conjugates, *S. aureus* type 8 capsular PS linked with ADH linker via random chemistry to *r*EPA (saccharide to protein ratio of 0.87) or DT (saccharide to protein ratio of 1.08) was more immunogenic in mice than the corresponding random conjugates with cystamine and SPDP (*r*EPA conjugate saccharide to protein ratio of 0.85, DT conjugate saccharide to protein ratio of 0.94). (Fattom et al., 1992; Fattom et al., 1995).

### 4.8 Streptococcus pneumoniae

Synthetic tetrasaccharides of PnPS 14 terminally conjugated to CRM_197_ using N-hydroxysuccinimide-activated adipic acid diester (average number of OS units per protein of 4) were more immunogenic in mice than when a diethyl squarate spacer (average number of OS units per protein of 4.8) was used (Mawas et al., 2002). Similar immunogenicity was obtained in young children when two bivalent pneumococcal conjugate vaccines were compared, consisting of serotype 6B and 23F OS conjugated to CRM_197_ directly or by a 6-carbon adipic acid spacer (Steinhoff et al., 1994).

### 4.9 Vibrio cholerae

Gupta et al. (Gupta et al., 1992) found that *Vibrio cholerae* O1 serotype Inaba deacylated lipopolysaccharides (LPS) (bimodal molecular weight population with two peaks at 13 kDa and 6 kDa) conjugated to cholera toxin (CT) were more immunogenic when obtained by random chemistry with ADH (saccharide to protein ratio of 0.8) than when produced by single-point attachment, using SPDP linkers (saccharide to protein ratio of 0.72). In another study, linkers of three different chain length (linkers extended by two (H_2_N(CH_2_)_2_NH_2_)- and six (H_2_N(CH_2_)6NH_2_)-carbon chains, respectively, compared to the use of an hydrazide linker (H_2_NNH_2_)) did not impact on the immunogenicity of *V. cholerae* O1 Ogawa serotype hexasaccharide-BSA selective conjugates tested in mice (Saksena et al., 2006).

## 5 Carrier protein

Theoretically, any protein can be used as a carrier protein. In addition to the ability to activate T-cells immune responses, important features to consider when choosing a carrier protein are purity and safety, manufacturability at industrial scale, and the possibility of being fully characterized by analytical methods. Additionally, a low likelihood of generating an antibody response to the carrier protein’s specific B cell epitopes can help to focus the immune response on the sugar hapten and allow for easy combination with other possible antigens with minimal immune interference. However, the possibility that the carrier protein originates from the pathogen of interest, thus also having a role as a protective antigen in addition to serving as a carrier, is an attractive possibility (Micoli et al., 2018).

Currently, five carrier proteins have been approved for use: diphtheria toxoid (DT), tetanus toxoid (TT), CRM_197_ (a non-toxic variant of diphtheria toxin), *Haemophilus* protein D (PD), and the outer membrane protein complex of serogroup B meningococcus (OMPC) (Pichichero, 2013; Micoli et al., 2018). TT and DT have traditionally been used because of safety data collected with tetanus and diphtheria vaccination. CRM_197_ does not require chemical detoxification, facilitating production and resulting in homogeneous preparations (Broker et al., 2011). Merck has used OMPC for its Hib conjugate vaccine (Donnelly et al., 1990) and GSK has used Hib-related protein D in a 10-valent pneumococcal conjugate vaccine (Forsgren et al., 2008). Other proteins have been used in conjugate vaccines under development, including *r*EPA as carrier for *S. aureus* types 5 and 8, *Salmonella* Typhi Vi, *Shigella* conjugate vaccines (Kossaczka et al., 1997; Fattom et al., 2004; Passwell et al., 2010) and several bioconjugates directly synthesized in *E. coli* (Wacker et al., 2014; Hatz et al., 2015; Riddle et al., 2016; Huttner et al., 2017). Investigating new carrier proteins is important due to factors such as pre-exposure and co-exposure to certain carriers leading to reduced anti-carbohydrate immune response and for a potential dual role as both a carrier and protective antigen. In this, case site-specific conjugation chemistries and glycoengineering are preferable to prevent detrimental effects on protein immunogenicity. Novel multivalent carrier systems, such as nanoparticles and mdOMV/GMMA, are being explored for their potential to present multiple copies of carbohydrate antigens and for their innate adjuvant properties (Micoli et al., 2018).

Below we report those studies in which different carrier proteins have been compared.

### 5.1 Candida albicans

Laminarin (Lam) from the brown alga *Laminaria digitata* was the model saccharide used for an immunogenicity study comparing different and novel carrier proteins for glycoconjugate vaccines against *Candida albicans*. Twenty-eight proteins from various pathogens were selected for recombinant expression in *E. coli* based on criteria such as lack of toxicity, solubility in physiological buffers, sufficient number of lysines for conjugation, and molecular weight between 40 and 100 kDa. The proteins were conjugated to Lam via reducing end, using the same active ester/protein molar ratio, and compared to a glycoconjugate using CRM_197_ as carrier (saccharide to protein ratio varied from 1.7 to 0.1 w/w). The pneumococcal protein spr96/2021 was shown to be a promising carrier inducing IgG titers significantly higher than CRM_197_-Lam (Tontini et al., 2016).

### 5.2 Clostridium difficile

In a recent study, selective chemistry was used to conjugate recombinant fragments of the toxins TcdA_B2 and TcdB_GT from *Clostridium difficile* to the PS PSII from the same organism (13 kDa). It was found that only the TcdB_GT conjugate was able to generate anti-PSII IgG levels in mice that were similar to those produced by a CRM_197_-PSII conjugate, with all conjugates bearing comparable saccharide to protein ratios (0.28–0.33 w/w). Both conjugates elicited anti-carrier IgG with toxin-neutralizing activity that were comparable to those of the non-conjugated proteins (Romano et al., 2014).

### 5.3 Escherichia coli

Conjugates were created by random carbodiimide-mediated coupling of ADH-derivatives of *E. coli* K100 PS respectively with TT (protein to saccharide ratio = 2.3 w/w) and horseshoe crab hemocyanin (HCH) (protein to saccharide ratio = 2 w/w). The *E. coli* K 100-HCH conjugate was more immunogenic than the *E. coli* K100-TT conjugate in mice (Chu et al., 1983).

### 5.4 B *Streptococcus*

GBS type III PS (460 kDa) conjugated to *r*CTB by random chemistry (saccharide to protein ratio of 0.78 w/w) induced higher IgG and IgA response in the lungs and vagina in mice after intranasal immunization, than with TT (saccharide to protein ratio of 0.8 w/w) as carrier protein (Shen et al., 2000).

In another study (Nilo et al., 2015b), GBS type V capsular PS activated by oxidation and randomly conjugated to CRM_197_ (saccharide to protein ratio of 1.9 w/w) or GBS67 (saccharide to protein ratio of 1.8 w/w), a pathogen-specific pilus protein with the dual role of T cell carrier for the PS and antigen, showed comparable immune responses in mice. Similarly, GBS type II capsular PS conjugated via reductive amination to another GBS-specific pilus protein, GBS80, by random conjugation (saccharide to protein ratio of 1.8 w/w) showed comparable immunogenicity than a random glycoconjugate using CRM_197_ (saccharide to protein ratio of 1.1 w/w) (Nilo et al., 2015a).

### 5.5 *Haemophilus influenzae* type b

While Hib OS (3–10 repeating units) conjugated to CRM_197_ or DT via reducing end produced the same primary response in infants 19–23 months of age, on boosting the CRM_197_ conjugate elicited higher antibody levels than the DT conjugate. Five months later the antibody levels induced by the CRM_197_ conjugate had declined faster, so that levels for both conjugates no longer differed significantly. In 12–16 months old infants, only the CRM_197_ conjugate elicited strong primary and secondary antibody responses (Anderson et al., 1985).

Some studies have investigated T cell epitope carriers (de Velasco et al., 1995; Könen-Waisman et al., 1999; Amir-Kroll et al., 2003; Avci et al., 2011). Falugi et al. (Falugi et al., 2001) used three recombinant carrier proteins (N6, N10, N19) consisting of strings of 6, 10 or 19 human CD4^+^ T cell epitopes from various pathogen-derived antigens, including TT and proteins from *Plasmodium falciparum*, influenza virus and hepatitis B virus. When conjugated to Hib OS (5.5 KDa) via reducing end, the helper effect of the carrier was proportional to its size with Hib-N19 eliciting an anti-Hib IgG response greater than Hib-CRM_197_ (Falugi et al., 2001).

In another mouse study, the use of TT or horseshoe crab hemocyanin (HCH), respectively as carrier proteins, was found to similarly affect the immunogenicity of random Hib conjugates made by cyanogen bromide activation with ADH and conjugation to the protein (Chu et al., 1983). However, when Hib conjugates, prepared with the same chemistry (Chu et al., 1983) using TT, HCH or Cholera Toxin (CT) as carrier proteins, were compared in rhesus monkeys (Schneerson et al., 1984), Hib-HCH was less immunogenic than Hib-TT in juvenile monkeys and Hib-CT more immunogenic than Hib-TT in infant monkeys.

### 5.6 Klebsiella pneumoniae

*Klebsiella* serotype 11 octasaccharides were conjugated to KLH (molar ratio saccharide to protein of 18) or BSA (molar ratio saccharide to protein of 5) carrier proteins and their immunogenicity was compared in mice. The two conjugates induced a comparable immune response in BALB/c mice, while only the KLH conjugate was able to induce an immune response in (CBA/N x C3H/HeN)F1 mice, possessing an immature set of B cells. This suggests that certain carrier determinants present in KLH, but not in BSA, are likely to be required for the induction of antibodies with OS-protein conjugates in animal models with immature B cells (Zigterman et al., 1985).

### 5.7 Neisseria meningitidis

Similarly to Hib glycoconjugates (Falugi et al., 2001), the polyepitope carrier N19 (saccharide to protein ratio of 0.4) exerted a stronger carrier effect than CRM_197_ (saccharide to protein ratio of 0.49) in mice when conjugated to MenC OS (MW around 4 kDa) (Baraldo et al., 2004). Indeed, after two immunizations, the N19-conjugate induced anti-MenC antibody and protective bactericidal antibody titers higher than those induced by three doses of the CRM_197_-conjugate. In addition, after three doses, lower amounts of N19-based conjugate induced levels of anti-MenC IgG antibodies significantly higher than those induced by the CRM_197_-conjugate (e.g., N19-MenC at 0.156 μg versus CRM_197_-MenC at 0.625 μg).

In another study in mice, 8 recombinant proteins from various pathogens were evaluated as carriers for meningococcal saccharide antigens in comparison to CRM_197_ for selective conjugate vaccines. Some of the proteins, such as the pneumococcal spr 96/2021 and spr1875 from pneumococcus, and Upec-5211 and Orf3526 from Extra Intestinal Pathogenic *E. coli* (saccharide to protein ratios of 0.1–0.6 w/w), showed good promise as carriers as the corresponding conjugates elicited in mice anti-meningococcal serogroup C IgG titers similar to those obtained with the CRM_197_-based glycoconjugate (MenC OS MW around 4 kDa). Men A, Men W, Men Y and Men X - spr96/2021 conjugates, after three doses, induced specific anti-Men IgG and bactericidal titers comparable to corresponding CRM_197_ conjugates (Tontini et al., 2016).

It is important to evaluate the effect of prior exposure to carrier proteins on the anti-carbohydrate response to detect potential immune interference in individuals who have previously received vaccines containing DT and TT carrier proteins. Comparing the immunogenicity of Men OS (MW around 4 kDa) conjugated to CRM_197_, DT, and TT using a selective chemistry (comparable saccharide to protein ratio of = 0.1—0.4 w/w), the three carriers were equally effective in inducing an immune response against the carbohydrate moiety in mice without prior immunization, for all the serogroups tested (A, C, W-135 and Y). However, using Men A as a model, CRM_197_ priming had a stronger ability to enhance the anti-carbohydrate response elicited by CRM_197_ conjugates or conjugates with the antigenically related carrier DT, while TT priming did not have any effect. Additionally, repeated exposure of mice to TT or CRM_197_ before immunization with the respective MenA conjugates resulted in a significant suppression of the anti-carbohydrate response with TT conjugate and only a slight reduction with CRM_197_ conjugate. The effect of carrier priming on the anti-MenA response of DT-based conjugates varied depending on the carbohydrate to protein ratio (Tontini et al., 2013).

### 5.8 *Salmonella*

*Salmonella* Enteritidis O-SP conjugates were similarly immunogenic in mice when coupled to *S.* Enteritidis flagellin monomers, polymers or CRM_197_ by random conjugation with no linker (Simon et al., 2011). Simon et al. proposed flagellin as the carrier protein for *Salmonella Enteritidis* O-SP, both being a virulence factor and protective antigens with the potential to achieve enhanced protection by the additive effect of anti-O-SP and anti-flagellin immune responses. Mice immunized with core polysaccharide-OPS (COPS) respectively conjugated to CRM_197_ or flagellin polymer by random chemistry with the same PS/protein ratio elicited similar anti-LPS IgG levels and were significantly protected from lethal challenge with wild-type *S.* Enteritidis (80%–100% vaccine efficacy) (Simon et al., 2011).

Conjugates of the 13 amino acids peptide non-natural pan DR epitope (PADRE) with the human milk oligosaccharide, lacto-N-fucopentose II or a dodecasaccharide derived from *Salmonella* Typhimurium O-SP induced high glycan IgG titers in mice, which were comparable to glycoconjugates employing human serum albumin (HSA) as the carrier protein (Alexander et al., 2000).

Studies comparing Vi conjugates against *Salmonella* Typhi, obtained by random chemistry with ADH linker and different carrier protein, CRM_197_ and TT (Micoli et al., 2011), or DT and *r*EPA (Cui et al., 2010) found no effect of the carrier protein on immunogenicity in mice. In another study, full length Vi (165 kDa) conjugates induced strong IgG responses after just one dose, regardless of the carrier protein used (CRM_197_, DT, or TT), and a second dose did not increase the response. Fragmented Vi (43 kDa) conjugated to CRM_197_ and DT (but not TT) resulted in lower anti-Vi IgG responses after the first dose compared with full-length Vi conjugates. However, after a second dose, IgG levels were similar to those induced by full-length Vi conjugates, regardless of carrier protein (Arcuri et al., 2017).

### 5.9 Shigella

Vaccination of healthy adults using O-SP of *Shigella sonnei* conjugated to succinylated *r*EPA (saccharide to protein ratio of 2.02 w/w) or to native (saccharide to protein ratio of 1.35 w/w) or succinylated (saccharide to protein ratio of 2.06 w/w) *Corynebacterium diphtheriae* toxin mutant CRM9 by random chemistry and ADH, gave the same anti-LPS IgG levels (Passwell et al., 2001). With *S. flexneri* 2a O-SP, the *r*EPAsucc conjugate (saccharide to protein ratio of 2.58 w/w) gave significantly higher antibody levels than the CRM9succ conjugate (saccharide to protein ratio of 2.58 w/w) (Passwell et al., 2001). The opposite result was obtained in mice, with *S. flexneri* 2a-CRM9 (saccharide to protein ratio of 1.70 w/w) being more immunogenic than *S. flexneri* 2a-*r*EPA (saccharide to protein ratio of 0.60 w/w) (Pavliakova et al., 1999).

### 5.10 Staphylococcus aureus

*Staphylococcus aureus* type 8 capsular PS conjugated via random chemistry to DT with SPDP linker (saccharide to protein ratio of 0.94 w/w) was more immunogenic in mice than when *r*EPA was used (saccharide to protein ratio of 0.85 w/w), but there was no difference when the linker was ADH (DT conjugate saccharide to protein ratio of 1.08 w/w, *r*EPA conjugate saccharide to protein ratio of 0.87 w/w) (Fattom et al., 1995).

### 5.11 Streptococcus pneumoniae

Pneumococcal type 3 PS conjugated by random chemistry to TT (saccharide to protein ratio of 1.24 w/w) induced higher PS-specific IgG antibody levels in mice than BSA (saccharide to protein ratio of 0.52 w/w) and DT (saccharide to protein ratio of 1.17 w/w) conjugates prepared by the same method (Beuvery et al., 1982). In another study, the total IgG and IgM antibody concentrations induced in mice by PnPS 14 conjugated by random chemistry to BSA (saccharide to protein ratio of 0.85 w/w) were higher than those induced by *Salmonella* Typhi flagellin conjugate (saccharide to protein ratio of 1.4 w/w) (van de Wijgert et al., 1991), obtained using the same carbodiimide conjugation chemistry. Comparing two tetravalent vaccines containing pneumococcal type 6B, 14, 19F and 23F PS in a double-blinded study of young infants, antibody concentrations elicited by PnPS-TT were generally higher than those elicited by PnPS-DT (Dagan et al., 1997). However, there was a significant booster response to each serotype in both the PnPS-TT and PnPS-DT groups using unconjugated PS 6 months after priming.

A genetically detoxified pneumolysin, pneumolysoid (PLD), was investigated as a carrier protein for pneumococcal capsular PS, serotypes 6B, 14, 19F, and 23F and compared to TT conjugates made by random activation. Pneumolysin is a toxin produced by all types of *S. pneumoniae* and is a major virulence factor. The PLD conjugates were shown to be equivalent to or better in terms of PS-specific responses than the TT conjugates and elicited high levels of pneumolysin-specific IgG able to neutralize pneumolysin-induced hemolytic activity *in vitro* (Michon et al., 1998).

Sigurdardottir et al. compared DT and TT as carrier for an octavalent (serotypes 3, 4, 6B, 9V, 14, 18C, 19F and 23F) pneumococcal random conjugates formulation, finding that serotype-specific immune responses depended on the carrier protein used: DT gave better primary responses to serotypes 3, 9V and 18C, whereas TT gave better response to serotype 4 (Sigurdardottir et al., 2002). Similar responses were induced to the other serotypes. Good booster IgG responses were obtained in all vaccine groups.

Chu et al., comparing TT with the carrier horseshoe crab hemocyanin (HCH) for random PnPS 6A glycoconjugate vaccines in mice, found that PnPS 6A-TT (saccharide to protein ratio of 0.59 w/w) induced higher anti PS specific IgG levels than PnPS 6A-HCH (saccharide to protein ratio of 0.36 w/w) (Chu et al., 1983).

## 6 Conclusion

Many animal studies have investigated the influence of saccharide chain length, saccharide to protein ratio, conjugation method and carrier protein on the immunogenicity of traditional glycoconjugate vaccines (Table 1). Although in some studies more than one vaccine variable is altered at a time and the effects can be saccharide-specific, the importance of these parameters in determining overall immunogenicity is evident and some general principles emerge. Few studies have been performed with the newer technologies such as bioconjugates, dmOMVs and MAPS. Lessons learned from traditional glycoconjugates may also be relevant for the design of these next-generation polysaccharide and OS-based vaccines.

Synthetic OS have facilitated careful study of the influence of saccharide chain length and saccharide density on the immunogenicity of glycoconjugates (Pozsgay et al., 1999; Mawas et al., 2002; Pozsgay et al., 2007), but they are generally small in size. Anti-saccharide responses in animals can potentially be induced by conjugates with short OS comprising only one repeating unit of the corresponding PS or even only fragments thereof (Alonso de Velasco et al., 1993; Benaissa-Trouw et al., 2001; Jansen et al., 2001; Mawas et al., 2002; Jansen and Snippe, 2004). However, human studies in infants evaluating Hib or pneumococcal conjugates that differ in the length of OS indicate that increased immunogenicity occurs with longer OS (Anderson et al., 1986; Pichichero et al., 1998).

The effect of saccharide length on immunogenicity is related to other parameters, in particular hapten loading and conjugation chemistry. The use of low MW OS may maximize the T cell help offered by the carrier protein, but such glycoconjugates may fail to develop functional anti-PS antibodies if key conformational epitopes on the PS are lost (Peeters et al., 1991; Pozsgay, 2000; Peeters et al., 2003). It is likely that a minimal chain length is required to induce a strong immunological response that varies for each saccharide type (Peeters et al., 1992; Chong et al., 1997).

Some studies do not report an influence of the saccharide to protein ratio on the immunogenicity of conjugate vaccines (Laferrière et al., 1997; Benaissa-Trouw et al., 2001). The effect of this parameter is strongly influenced by saccharide size and conjugation chemistry. In general, lower saccharide to protein ratios increase the immunogenicity of native PS conjugated by random chemistry. This may be related to the large size and high degree of cross-linking of such conjugates, with a critical amount of protein required to stimulate an optimal T-dependent response. With terminally linked OS, the optimal saccharide to protein ratio depends on saccharide length, but generally immunogenicity is directly correlated to the saccharide to protein ratio. This is likely to result from better cross-linking and activation of saccharide-specific B cells with increased OS loading. Nonetheless, with high saccharide-to-protein ratios, essential carrier epitopes may be hidden from the immune system, hindering recognition of the conjugate vaccine as a T-dependent antigen (Pozsgay et al., 1999; Peeters et al., 2003).

The choice of conjugation strategy and linker has an important effect on conjugation efficiency, saccharide to protein ratio and vaccine size, resulting in an impact on immunogenicity (Beuvery et al., 1983; Carmenate et al., 2004). Different moieties of the saccharide and different amino acids of the protein can be involved in the conjugation process influencing which epitopes are exposed. In general, with short OS, selective chemistries are better than random ones and the converse applies with long PS. Linkers can differ in length and flexibility affecting the distance between saccharide and protein and the access of antigen binding sites to surface immunoglobulin on B cells.

Most animal studies indicate that the choice of carrier protein has an impact on conjugate vaccine immunogenicity. Overall, it is not straightforward to draw conclusions about which protein carrier has the optimal impact on the immunogenicity of glycoconjugate vaccines. This parameter is likely to be antigen-specific and interdependent with other factors such as conjugation chemistry, the presence of a spacer, and the size and degree of conjugation of the saccharide. Furthermore, the valency of the administered glycoconjugate (i.e., monovalent vs. multivalent) is likely to play a role, particularly in clinical settings where immune interference caused by (pre)exposure to the same carrier protein can result in a positive or negative effect on the immune response to the conjugated carbohydrate antigen.

Predicting successful vaccine design strategies for humans, especially infants, is not straightforward. Most of the published data on the influence of variables of conjugation on vaccine immunogenicity comes from animal studies. Clinical trials investigating conjugates differing in only one parameter are rare (Anderson et al., 1989; Steinhoff et al., 1994; Daum et al., 1997; Kossaczka et al., 1999; Passwell et al., 2001) and only a few studies have evaluated whether the results obtained on animals are reproducible in humans (Anderson et al., 1986; Pichichero et al., 1998). The determination of vaccine structure-immunogenicity relationships for humans, and especially for infants, is limited by ethical issues related to testing candidate vaccines in humans that are poorly immunogenic in animals, and by the cost of clinical trials. Good animal models are important for prioritizing which vaccines move into clinical trials. For example, Siber et al. showed that a guinea pig model could successfully predict the immunogenicity of Hib conjugate vaccines in humans (Siber et al., 1995).

Furthermore, animal studies usually focus on the influence of conjugation variables on the immediate immune response. Studies investigating the effect of parameters on antibody persistence and memory response are also needed. Current licensed conjugate vaccines are often characterized by a marked decline in antibody levels over time, particularly in infants, with multiple primary doses and/or boosters required to ensure protection. Improving the longevity of the immune response would increase the benefit-cost ratio of conjugate vaccines.

In conclusion, conjugation parameters are strongly interconnected in determining vaccine efficacy and the best candidate glycoconjugate vaccines will result from an optimal combination of each of these variables. Studies that use a matrix approach to examine the influence of different combinations of variables may provide the fastest route to identifying the most promising vaccines.
